# Adolescents' Experiences With Sequencing for Genetic Predisposition in Pediatric Cancer: A Quantitative Study

**DOI:** 10.1002/pon.70458

**Published:** 2026-04-07

**Authors:** S. B. B. Bon, R. H. P. Wouters, J. J. Bakhuizen, M. M. van den Heuvel‐Eibrink, H. Maurice‐Stam, M. C. J. Jongmans, M. A. Grootenhuis

**Affiliations:** ^1^ Princess Máxima Center for Pediatric Oncology Utrecht the Netherlands; ^2^ Department of Psychiatry Amsterdam University Medical Center University of Amsterdam Amsterdam the Netherlands; ^3^ Department of Genetics University Medical Center Utrecht Utrecht the Netherlands; ^4^ Division of Child Health Wilhelmina Children's Hospital University Medical Center Utrecht Utrecht the Netherlands

**Keywords:** adolescents, cancer, cancer predisposition, genetic counseling, oncology, pediatric oncology, psycho‐oncology

## Abstract

**Background:**

Germline sequencing is increasingly integrated into pediatric oncology, yet data on adolescents' experiences with genetic testing remain limited.

**Aims:**

To explore adolescents' knowledge, expectations, hopes, worries, satisfaction, and regret related to germline sequencing in the context of pediatric cancer.

**Methods:**

Adolescents with cancer (aged 12–18 years) who underwent cancer predisposition gene panel sequencing (143 genes) completed questionnaires at two time points: after providing consent and after receiving results. The questionnaires assessed genetics knowledge, expectations, hopes, worries, satisfaction, and decisional regret.

**Results:**

Of 109 eligible adolescents, 46 completed the first measurement, and 38 completed the second. Before disclosure of results, participants on average answered 49% of the genetics knowledge statements correctly. Nearly all hoped their participation would benefit future patients, and many expressed personal hopes, such as understanding why they developed cancer and whether their future offspring might be at risk. Worries were common, with fear of cancer recurrence being the most frequently reported concern, followed by concerns about risks to future offspring. Adolescents who received a negative test result (36 out of 38 of our sample), reported high satisfaction with study participation and low levels of regret.

**Conclusions:**

Adolescents with cancer generally report positive experiences with germline sequencing, despite limited genetics knowledge and prevalent worries. Adolescents are not only concerned about their own health, but also about the health of family members, future offspring, and potential future patients.

## 1 Background

Up to 10% of children with pediatric cancer have an underlying genetic cancer predisposition [1]. Identification of these genetic conditions is an important component of care for children with cancer, as it may lead to surveillance for second tumors, counseling of family members about their risks, and in some cases treatment adjustments [2, 3]. Therefore, some advocate for the implementation of germline sequencing of a broad range of cancer predisposition genes as part of the diagnostic process for all children with cancer [4].

A substantial proportion, approximately 50%, of children with cancer are diagnosed between 10 and 18 years old [5, 6, 7]. Recent studies have shown that, just like with other aspects of their care, adolescents desire to be involved in decision‐making regarding germline genetic testing [8, 9]. To fulfill this need, an informed consent process that is tailored to the capabilities and views of adolescents is crucial [10]. Studies among the general population report that adolescents often have limited or inaccurate understanding of genetics [11, 12]. Adolescents might, for example, have difficulties understanding how DNA aberrations contribute to the development of diseases [12]. Studies specifically into cancer genetics suggest that adolescents' knowledge about this subject is often limited as well, even in families that are affected by hereditary cancer [13, 14].

An important prerequisite for an effective informed consent process is that it addresses topics that are meaningful to adolescents in making an informed decision [9]. Previous publications suggest that adolescents and young adults are motivated to participate in genetic testing to protect their family members, discover the cause of their cancer, and enable them to plan for the future [15]. Additionally, helping future patients has been reported to be an important motivating factor for adolescents to participate in genetic research [16, 17]. Although few studies have examined long‐term psychological outcomes following germline testing, existing studies generally report few major negative psychological impacts within the studied follow‐up periods [18]. Nevertheless, adolescents may experience concerns, such as the implications of genetic testing for their family members and future insurability [19].

As there is limited research on the perspectives of adolescents with cancer undergoing germline sequencing outside of selected groups, such as patients with a family history of cancer or who underwent paired tumor‐germline sequencing to identify additional treatment options, we aimed to explore adolescents' experiences with broad cancer predisposition sequencing a few months after their cancer diagnosis. The adolescents were undergoing genetic sequencing as part of a research project and had the option to participate in a psychosocial add‐on study. We examined the knowledge levels of adolescents and explored their expectations, hopes, worries, and satisfaction with the sequencing study, as well as feelings of regret with sequencing participation. Additionally, we explored whether demographical and medical characteristics of adolescents are associated with lower knowledge levels and worries regarding genetic sequencing.

## 2 Methods

Between December 2021 and January 2022, a psychosocial study (REFLECT) was conducted among families participating in a Dutch nationwide cancer predisposition sequencing study in pediatric oncology (PrediCT), that was open to all newly diagnosed patients during that period; all adolescents who participated in the PrediCT sequencing study during this time were invited to take part in the REFLECT psychosocial study [20]. In this paper, the results of the questionnaire study conducted among adolescents aged 12 to 18 will be discussed. The results of other studies from this project, a qualitative interview study among the same age‐group, and a questionnaire study among parents, have been published elsewhere [21, 22].

### 2.1 PrediCT Sequencing Study

All newly diagnosed adolescents (aged 12–18 years) were invited for the cancer predisposition sequencing study by a physician‐researcher approximately five months after their cancer diagnosis, with approval from their treating pediatric oncologist. Recruitment was delayed in adolescents experiencing acute complications (e.g., recent relapse or ICU admission). Adolescents with a cancer predisposing condition known prior to the cancer diagnosis were excluded. Adolescents referred for clinical genetic evaluation after cancer diagnosis, could participate if no causative predisposition was identified by the clinical geneticist [20].

In accordance with Dutch legislation, dual consent was required from both the adolescent and their parents for participants aged 12–15. For adolescents aged 16 and older, consent was provided solely by the adolescent. Families were initially informed about the sequencing study by the physician‐researcher via phone. A follow‐up meeting at the outpatient clinic provided further information on key topics, including early disease detection, potential insurability issues, and living with a genetic predisposition.

In the sequencing study, 143 cancer predisposition genes were analyzed, pathogenic and likely pathogenic variants were disclosed to families by a clinical geneticist or clinical geneticist in training. More information about the sequencing study can be found elsewhere [20].

### 2.2 REFLECT Questionnaire Study

Recruitment for the REFLECT questionnaire study took place at the same time as the informed consent procedure for the sequencing study (PrediCT). Adolescents were informed separately about the questionnaire study, and a distinct informed consent letter was used. Participation in the questionnaire study was optional and required participation in PrediCT, meaning that all adolescents who participated in the questionnaire study underwent cancer predisposition sequencing, while not all adolescents who underwent sequencing participated in REFLECT.

The questionnaire study consisted of two measurements. At the first measurement (T1), adolescents had recently given consent for the sequencing study. The second measurement (T2) was administered approximately 1 month after the final sequencing results were disclosed. Exclusion criteria for the questionnaire study included insufficient proficiency in Dutch and a diagnosis of a genetic variant that caused the adolescent's cancer.

### 2.3 Measures

Both measurements consisted of several questionnaires developed by a multidisciplinary team with expertise in pediatric psycho‐oncology, clinical genetics, ethics, and pediatric oncology. Where relevant, adaptations were informed by questionnaires developed in a concurrent study conducted at the University of New South Wales (UNSW), with the aim of facilitating future cross‐national comparisons.

The measurements combined items derived from existing validated instruments (which were adapted to fit the context of adolescents with cancer undergoing germline sequencing) with items specifically developed for this study, based on expert knowledge and relevant literature. For transparency, the origin of each item included in Tables 3 and 4 is explicitly indicated. Items for the adolescent questionnaires were developed in parallel with those for the adjacent study among parents [22]. The Dutch Childhood Cancer Association (VKKN) assisted in creating the questionnaires, and several international experts were consulted. Additionally, the questionnaires were piloted with three healthy adolescents, whose feedback was used to improve readability.

An online platform was used to administer the questionnaires (www.hetklikt.nu). Adolescents received an email stating that the questionnaire was available, if they did not complete it, they received two more emails and finally a phone call by one of the researchers.

### 2.4 Knowledge

First, adolescents were asked whether they were satisfied with their knowledge when making the decision about participating in the sequencing study. For this purpose, a single statement to which adolescents could respond on a five‐point Likert scale was developed (completely disagree to completely agree). To measure knowledge shortly after consent for sequencing (T1), the Precision in Pediatric Sequencing Knowledge Questionnaire was translated and modified (PiPseqKQ) [25]. Modifications were made to ensure alignment with the context of the current study and with the aforementioned study conducted at the UNSW. The modified version consisted of 23 factual statements to which adolescents could respond with, “true”, “false” or “not sure”. A total score was calculated based on the number of correctly answered statements. The internal consistency of the questionnaire was acceptable (Cronbach's alpha 0.86).

### 2.5 Hopes and Expectations

During the first measurement, adolescents were presented with 16 statements about their hopes and experiences related to deciding on participation in genetic testing. Where available, items were adapted from previous literature [17, 24], and remaining items were developed specifically for this study. This questionnaire used a five‐point Likert scale, ranging from completely disagree to completely agree, responses were dichotomized into disagree and agree or completely agree [17, 24]. In addition, participants were asked how likely they thought it would be that they would have a predisposition and how likely they thought it would be that they would obtain benefits for themselves from genetic testing. Adolescents could respond with very likely (> 90% chance), likely (75%–90%), moderately likely (50%–74%), somewhat likely (25%–49%), very unlikely (< 10%) or no chance.

### 2.6 Worries

To explore worries of adolescents, two measures were used. At the first measurement, six worry items based on previous literature were used in the same fashion as the previously described five‐point Likert scale [17, 24]. Next to these questions, an adaptation of the Psychosocial Aspects of Hereditary Cancer (PAHC) questionnaire was used at both measurements. This questionnaire was developed to assess germline testing related distress in adult oncology [23]. The items of the PAHC were altered to match the reading level and context of adolescents with cancer, several questions were omitted because they were deemed irrelevant in this specific context. At the second measurement, two versions were used, depending on whether adolescents received a positive (11 items) or negative test (8 items) result. Adolescents could respond to the items with “not at all,” “a little,” “quite a bit,” or “very much”. In accordance with the original PAHC questionnaire, if participants indicated being worried ‘quite a bit’ or ‘very much’ on any of the queried items, this was taken to imply that they may experience distress related to the sequencing process. For each measurement, a dichotomous score was calculated to determine whether worries were present on one or more topics. The internal consistency of the questionnaire during the first measurement was acceptable (Cronbach's alpha 0.74). The Cronbach's alpha for the second measurement questionnaire was 0.84 in adolescents who received a negative test result. For adolescents with a positive test result this value could not be calculated due to the small numbers.

### 2.7 Satisfaction and Regret

At the second measurement, after adolescents received their test results, they completed questionnaires on their satisfaction with undergoing genetic testing and their test results and regret regarding their decision to participate. Questions regarding satisfaction were based on previous literature or developed for this study and used a five‐point Likert scale in the same fashion as described above [17, 24]. In addition, regret was measured using a Dutch translation of the Decisional Regret Scale [26, 27, 28]. In accordance with the original scale, it consisted of 5 items, each using a Likert scale ranging from 1 to 5. Out of these items, a sum score was calculated between 0 (meaning no regret) and 100 (meaning maximal regret). In addition, the classification system as proposed by Becerra‐Perez et al. was used to categorize regret scores into no (0), mild (5–35) and moderate to strong regret (> 35) [29].

### 2.8 Demographical and Medical Characteristics

Sociodemographic data, including information about the parents' educational level and marital status, were obtained from the KLIK database, which is used both for clinical and research purposes. In cases where this information was missing, one parent was asked to complete the missing details. Medical variables, such as diagnosis, were obtained from the database of the PrediCT study.

Based on previous studies, the associations of gender, age, prior genetic exposure and parental educational level with knowledge levels and worries were assessed [25, 30]. Based on expert opinion, diagnosis group was added in our analysis.

### 2.9 Statistics

To summarize adolescent's background characteristics and the outcomes of the questionnaire, descriptive statistics were used. *T*‐tests, ANOVA, and linear regression were used to assess associations of demographical and medical characteristics with adolescent's score on the knowledge questionnaire. Univariate binary logistic regression and Chi‐square tests were used to assess associations of demographical and medical characteristics with whether adolescents experienced worries as measured by the adapted PAHC questionnaire. Furthermore, Chi‐square testing was used to assess differences on individual worries by gender. Results of adolescents who only completed part of the measurements are reported for the items that they did complete.

## 3 Results

**FIGURE 1:**
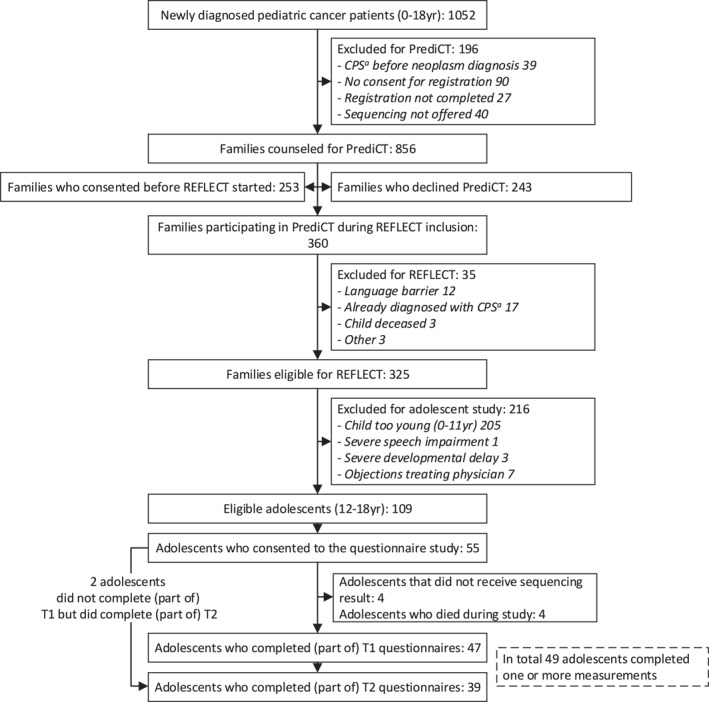
Flowchart of study inclusion.

In total, 109 adolescents were eligible and approached for this questionnaire study, 55 adolescents provided consent. Although not systematically asked and analyzed, we learned from adolescents who expressed their reasons for not participating in the questionnaire study that common factors included the burden of cancer treatment, sometimes combined with the demands of other studies they were involved in, or simply a lack of interest in this particular study. The first measurement of the questionnaire study was completed by 46 adolescents. 47 adolescents could complete the second measurement. Three of these adolescents had a positive test result, of which two completed the second measurement. To protect the privacy of these two adolescents, their experiences will be briefly summarized. Of the 44 adolescents with a negative test result 36 completed the second measurement. Four participants were deceased or in a terminally ill state at follow‐up; reasons for non‐completion among the remaining participants with a negative test result (*n* = 4) are unknown. In total, 49 adolescents completed part of either or both the first and/or second measurement. A detailed overview of the inclusion and response can be found in Figure 1. Table 1 shows the characteristics of the participating adolescents and their families, the characteristics of all participants of the sequencing study can be found in a separate publication [20].

**TABLE 1:** Participant characteristics.

Adolescent characteristics (*N* = 49)	Number (%) or mean [SD]	Missing
Male gender	53.2%	
Female gender	46.8%	
Age (years)	15.4 [1.91]	
Diagnosis group		
Hematological malignancy	36.5%	
CNS tumors	34.6%	
Non‐CNS solid tumors	28.8%	
Referred for genetic consultation^a^	34.6%	
Parental situation		
Together	90.0%	
Not together	6.1%	
Widowed	4.1%	
Parents born in The Netherlands	88.8%	
Time since diagnosis at first measurement (T1) (months)	8.0	
Time since diagnosis at second measurement (T2) (months	16.4	
Highest education of (either) parent^b^		6.1% (*n* = 3)
Low	6.1%	
Middle	26.5%	
High	61.2%	

^a^ Families who were referred for evaluation by a clinical geneticist could still participate in the sequencing study and the subsequent questionnaire study unless a cancer predisposing condition was identified during diagnostic testing.

^b^ Educational level defined according to Statistics Netherlands (CBS, Centraal Bureau voor de Statistiek), 2016: low educational level = no education, primary school, lower secondary education; middle educational level = upper secondary education, pre‐university education, intermediate vocational education; high educational level = higher vocational education, university.

### 3.1 Knowledge About DNA Sequencing

Most adolescents (62.6%) reported that they were satisfied with their level of genetic knowledge at the time of providing consent for the sequencing study. 10.4% were not satisfied with their knowledge and the other adolescents (27.1%) responded neutral.

On the knowledge questionnaire, adolescents scored on average 48.0% of the statements correctly (SD 21.3%, scores ranged from 0% to 87.0%). Adolescents frequently opted for the “not sure” option; on average they have chosen this option in 39.9% of the statements.

The topics that were most frequently misunderstood included the comparison of germline and tumor DNA in the analysis for cancer predisposing conditions (0% correct), the possibility of identifying mutations in genes unrelated to cancer (6.3% correct), and the role of sequencing results in guiding initial treatment of childhood cancer (10.4% correct). Scores on each specific statement can be found in Table 2.

**TABLE 2:** Knowledge about sequencing.

	Correctly answered%	Not correctly answered%	Not sure %	
Total knowledge score (*N* = 47)	48.0	11.4	39.9	
Half of your genes were inherited from your mother and half from your father (true)	79.2	10.4	10.4	
Our genes are located on our chromosomes which are made up of DNA (true)	52.1	6.3	41.7	
A complete set of a person's DNA is called a genome (true)	31.3	4.2	64.6	
Our genes tell our cells how to develop and function properly (true)	54.2	8.3	37.5	
Changes in the genes that make them faulty can affect how cells function and cause health problems (true)	70.8	2.1	27.1	
All changes in genes cause health problems (false)	75.0	4.2	20.8	
A person with a genetic (inherited) predisposition for a health problem will always get the problem (false)	89.6	2.1	8.3	
If a parent has a faulty gene, they can pass this gene fault to their child (true)	83.3	6.3	10.4	
If a person is the carrier of a cancer faulty gene, it means that they have cancer (false)	70.8	6.3	22.9	
A person's genes can influence if they will experience side effects (bad reactions) or benefits from a medication (true)	43.8	2.1	54.2	
A disease is only heritable if more than one family member is affected (false)	52.1	14.6	33.3	
Most childhood cancers are not inherited. They are caused by DNA changes that happen during the child's life (true)	47.9	6.3	45.8	
Only a small percentage of cancer genes have been found and as a result, the genetic causes of most cancers remain unknown (true)	56.3	6.3	37.5	
Genomic testing for cancer compares the DNA of cancer cells to the DNA of non‐cancer cells (false)	0.0	50.0	50.0	
Genomic testing can find gene faults that may be the cause of a person's cancer (true)	50.0	8.3	41.7	
Genomic testing cannot find gene faults that may affect how fast a cancer grows or if it is likely to spread to other parts of the body (true)	18.8	27.1	54.2	
Genomic testing can find changes in genes that are not known to cause any disease (true)	31.3	4.2	64.6	
It is not always clear if a change in a person's genes is a gene fault that is causing their cancer or if it is a normal change not related to a health problem (true)	41.7	8.3	50.0	
Genomic testing cannot find faults in genes that are known to cause health problems other than cancer (true)	6.3	37.5	56.3	
Gene faults found by genomic testing in genes that are not related to the cancer being tested are known as incidental findings (true)	47.9	0.0	52.1	
Incidental findings may also provide information about present and future health risks of other family members (true)	47.9	10.4	41.7	
Genomic testing that is done in children at diagnosis will commonly guide initial treatment (false)	10.4	31.3	58.3	
Genomic testing may find that a person has an increased chance of developing a health problem in the future (true)	60.4	4.2	35.4	

*Note:* True: Statement was true in the context of the sequencing studyFalse: Statement was not true in the context of the sequencing study. Adolescents were asked to answer the questions with the context of the current germline sequencing study in mind.

There were no significant differences in overall knowledge scores based on gender, parental educational level, diagnosis group and whether adolescents had been previously referred to a clinical geneticist. Only the age of adolescents was significantly associated with knowledge scores (*B* = 1.1, *p* = 0.003); knowledge increased approximately 1.1 point per year of age. An overview of all results can be found in Supporting Information S1.

### 3.2 Hopes and Expectations of Cancer Predisposition Sequencing

Out of a list of potential hopes that one might have regarding the outcomes of genetic sequencing for cancer predisposition, the following hopes were embraced by a majority of respondents: helping doctors and scientists to learn more (91.5%), finding cures for future patients (89.4%), providing information about ones cancer (78.7%), allowing screening for future disease (68.1%), learning if future offspring could become ill (59.6%), and learning if siblings could become ill (57.5%). It is worth noting that a substantial group of participants indicated that they hoped that germline sequencing would increase their chances of being cured and/or increase their treatment options (44.7% and 36.2%, respectively), even though they had been informed during the consent procedure that this was not to be expected. Thirty‐four percent of the adolescents did not expect any benefit from the sequencing. Table 3 provides an overview of the most common hopes, sorted from most to least common.

**TABLE 3:** Hopes and worries after consent for sequencing.

Hopes^a^ (*N* = 46)	Agree or completely agree %
I Hope it will help doctors and scientists learn more about the genes involved in cancer^c^	91.5
I Hope it will help find cures for future patients^c^	89.4
I Hope it will help provide information to me and the doctor about my cancer^c^	78.7
I Hope it will allow to screen for or prevent other conditions that I could develop^d^	68.1
I Hope to find out if my future children could become ill^e^	59.6
I Hope to find out if my siblings could become ill^e^	57.5
I Hope to find out if other family members could become ill^e^	53.1
I Hope it will help me to understand a possible cause for my disease^d^	51.0
I Hope it will increase my chance of being cured^c^	44.7
I Want to know as much as possible^e^	44.6
I Hope to gain more (general) genetic information about myself^c^	38.3
I Hope that doing this testing will provide me with peace of mind^c^	38.2
I Hope it will give me a greater number of treatment options^c^	36.2
I Do not expect any benefit to me or my family from this research^c^	34.0
Our doctor recommended the study^c^	25.6
Participating in this research study gives me hope^c^	23.4

**Worries from adapted PAHC** ^b^ **(*N* = 47)**	**Quite a bit or very much %**
Are you worried about the chance that you get cancer again?^f^	31.3
Are you worried about the chance that family members will get cancer?^f^	22.9
Are you worried about what it will be like to know that you have a predisposition?^f^	18.8
Are you worried about the impact of DNA results when you want to have children yourself in the future?^f^	25.0
Are you worried about having a genetic predisposition for cancer?^f^	16.7
Do you feel insecure about the future?^f^	16.7
Do you feel tense?^f^	12.5
Are you worried about possible extra check‐ups or other measures based on the test results?^f^	6.3
Are you worried about the impact of the test results on your own daily life (at school, at home, with friends or with hobbies)?^f^	6.3
Do you feel anxious?^f^	4.2
Do you feel depressed?^f^	0.0

**Other worries** **^a^** **(*N* = 46)**	**Agree or completely agree %**
I Worry that a potential predisposition might make it more difficult for me to live an untroubled life^c^	17.0
I Worry that I may learn information about my family's DNA that would be stressful or cause anxiety†‡	17.0
I Worry that I may learn information about my DNA that would be stressful or cause anxiety^c^	12.7
I Worry that the results may take a long time to come back^c^	6.4
I Worry that the information learned could hurt my family's ability to get insurance^c^	4.2
I Worry that the information learned in this study will not be kept private^c^	0.0

^a^ This questionnaire used a five‐point Likert scale ranging from (completely) disagree to (completely) agree. The two most rightward options were counted as agree.

^b^ This questionnaire used a four‐point scale (not at all, a bit, quite a bit, very much). Quite a bit and very much are considered to indicate worries [23].

^c^ Adapted from items used in the study by Marron et al. [17].

^d^ Adapted from items used in the study by Fernandez et al. [24].

^e^ Study‐specific items, some developed in collaboration with UNSW.

^f^ Adapted from the Psychosocial Aspects of Hereditary Cancer (PAHC) questionnaire [23].

**TABLE 4:** Satisfaction and worries after sequencing results disclosure.

Satisfaction among adolescents with positive and negative test results^a^ (*N* = 38)	Agree or completely agree %
I Am glad I chose to participate in genetic testing^c^	100
I Am satisfied with the overall process of genetic testing^e^	80.5
I Am reassured by the test results^e^	73.2
Participating in genetic testing taught me something about predisposition and genetics (in general)^e^	48.8
Participating in genetic testing gave me peace of mind^c^	48.8
I Feel that I helped future patients by participating in genetic testing^e^	43.9
Participating in genetic testing taught me something about cancer (in general)^e^	31.7
Participating in genetic testing increased my chance of being cured^c^	17.1
Due to the test result I'm able to better understand why I developed cancer^d^	12.2
I Am disappointed with the test results we received^c^	4.9
I Expected more of the test results^e^	4.9

**Worry questions from adapted PAHC among adolescents with negative test result** **^b^** **(*N* = 37)**	**Quite a bit or very much %**
Are you worried about having a genetic predisposition for cancer?^f^	2.5
Are you worried about the impact of DNA results when you want to have children yourself in the future?^f^	7.5
Do you feel anxious?^f^	0.0
Do you feel tense?^f^	0.0
Do you feel depressed?^f^	0.0
Do you feel insecure about the future?^f^	2.5
Are you worried about the chance that you get cancer again?^f^	20.0
Are you worried about the chance that family members will get cancer?^f^	5.0

^a^ This questionnaire used a five‐point Likert scale ranging from (completely) disagree to (completely) agree. The two most rightward options were counted as agree.

^b^ This questionnaire used a four‐point scale (not at all, a bit, quite a bit, very much). Quite a bit and very much are considered to indicate worries [23].

^c^ Adapted from items used in the study by Marron et al. [17].

^d^ Adapted from items used in the study by Fernandez et al. [24].

^e^ Study‐specific items, some developed in collaboration with UNSW.

^f^ Adapted from the Psychosocial Aspects of Hereditary Cancer (PAHC) questionnaire [23].

Adolescents were questioned about how likely they thought it would be that a cancer predisposition would be identified. Most adolescents (54%) estimated the likelihood to be less than 25%, while 15.2% believed the chance to be higher than 75%. The remaining participants provided estimates between these ranges.

### 3.3 Sequencing Related Worries

A list of 18 worries was presented to adolescents after they gave consent for sequencing (T1), of which 11 were part of the adapted PAHC questionnaire. None of these 18 worries was experienced by a majority of adolescents. At the same time, 49% of the adolescents indicated to be quite or very much worried about at least one topic. The most common worries include: worries about getting cancer again (31.3%), worries about the impact of DNA results when they want to have children themselves (25%), and worries about family members getting cancer (22.9%). We only found a significant difference in one of the worry questions based on gender: female adolescents more often worried than males that they would receive information about their family that would cause stress or anxiety (27.3% vs. 4.2%, *p* = 0.029). Table 3 shows all questions and the percentages of adolescents who expressed worry.

To assess whether certain subgroups were more at‐risk to experience psychosocial distress related to sequencing, we looked at the group of participants (43.8%) who indicated that they were quite worried or verry worried about at‐least one of the items on the PAHC list. Being very/quite worried was not significantly associated with gender, diagnosis group, being referred to clinical genetics, parental educational level and age. An overview of these results is presented in Supporting Information S1.

### 3.4 Experiences After Disclosure of Sequencing Results

All 38 adolescents indicated that they felt glad about their decision to participate in the sequencing study, and the majority (80.5%) reported being satisfied with the procedures of the sequencing study. Other commonly reported positive feelings were being reassured (73.2%) or experiencing peace of mind because of their participation (48.8%) and learning something about genetics (48.8%) and cancer (31.7%). Almost half of the participants felt that their participation had helped future patients (43.9%). Responses to all satisfaction questions can be found in Table 4.

Of the 36 adolescents who received a negative sequencing test result, 22.2% reported no regret (score 0), 66.7% reported mild regret (score 5–35), and 11.1% reported moderate to strong regret (score > 35). Most adolescents (71%) agreed or strongly agreed that participating in the study was a wise decision, and 87% agreed or strongly agreed that they made the right decision. Furthermore, 92% agreed or strongly agreed that they would make the same choice again. In contrast, only 3% agreed or strongly agreed that the decision caused harm, and 5% agreed or strongly agreed that they regretted making the decision at all.

After receiving a negative sequencing result, 25.0% of the adolescents reported worries. Most of the worries adolescents reported were on “getting cancer again” (20%), other worries were only reported in small minorities of the respondents. A complete overview of these worries can be found in Table 4. Being very or quite worried on one or more items was not significantly associated with demographic and medical characteristics. An overview of these results is presented in Supporting Information S1.

The two adolescents who received a positive test result reported several worries on the PAHC questionnaire. Like their peers with a negative test result, they expressed satisfaction with their decision to participate in the study, with regret scores of 10 and 35, both indicating mild regret.

## 4 Discussion

In this study, the experiences of adolescents with germline sequencing in pediatric oncology are described. We observe that understanding genetic concepts is challenging for many adolescents, including how genetic testing relates to their cancer treatment. Nearly half of the adolescents reported worries related to genetic sequencing after giving consent. After receiving negative test results, this proportion was one quarter. The most common worry among these adolescents was the fear of developing cancer again. Adolescents were satisfied with their participation in sequencing, and most showed none or little regret after the results were disclosed. In summary, the adolescents with cancer that participated in our study reported mostly positive experiences with sequencing. Nevertheless, several challenges remain that will be discussed in more detail.

Understanding the specific consequences of genetic testing is a key element of informed consent [31]. We found that adolescents on average scored less than half of the true/false statements presented to them correctly. The scores of the adolescents in this study are comparable to those of the parents who participated in our study and those of the (young) adult survivors and parents in the study that developed the knowledge questionnaire [22, 25]. It should be noted however that the PIPseqKQ was designed to get an impression of the general level of knowledge about genetics [25], not to measure whether individuals had sufficient understanding to make an informed decision regarding genetic testing. Nevertheless, participants' scores on specific items of the knowledge questionnaire can be highly informative and useful. For the adolescents in this study, the scope of the genetic testing and analysis were among the most difficult subjects to grasp. For example, many participants did not know (or at least were not certain) as to whether genetic conditions unrelated to cancer could be found and to what extend germline predisposition sequencing provides information about the tumor DNA. Educating participants about these topics is challenging, especially because the bandwidth of genetic sequencing and its potential findings is highly contingent on how the testing procedure and bioinformatics pipeline is designed [32]. Yet, more clarity is also necessary to prevent disillusionment and false reassurance further down the line. Interestingly, many adolescents reported feeling satisfied with their level of genetic knowledge, despite their relatively low scores. One possible explanation is that adolescents may overestimate their understanding of genetics, as has also been reported in parents [33, 34].

Previous studies in pediatric oncology have shown that many participants in sequencing studies have high hopes for positive outcomes and that most participants view their participation positively, even if their initial hopes are not fully met [16, 17, 35]. In this study, many adolescents expressed hopes that the sequencing study would result in useful information regarding their own or their family's health. At the same time, the majority of the adolescents perceived their chances of having a cancer predisposing condition as low and one third did not expect any personal benefit from their participation in sequencing. This combination of high hopes, for example hopes that genetic testing would benefit their own health, their offspring and family members, alongside more modest expectations suggest a distinction in what adolescents hope for and what they realistically anticipate. This distinction might contribute to the high satisfaction that adolescents report, even though they report that the hopes they had were not fulfilled. Consistent with the high overall satisfaction, most adolescents with a negative test result reported no or mild regret, indicating that, while some regret was present, it did not detract from their overall satisfaction. A previous study following parents after genome‐wide sequencing showed a comparable pattern of high hopes paired with realistic expectations. Despite initial disappointment when no diagnosis was found, most parents reported no or mild regret, similar to the adolescents in our study [36]. As highlighted in previous studies, a negative test result is not necessarily experienced as straightforward ‘good news’. Families are faced with the task of interpreting and giving meaning to such results, which may involve uncertainty about future risks and the limitations of current knowledge [37, 38, 39].

Helping science and future families faced with pediatric cancer are important reasons for adolescents with cancer to participate in research, as also found in this study [16, 17]. At the second measurement, less than half of the adolescents with a negative test result reported feeling that they had actually contributed. This finding highlights an opportunity to better inform adolescents about how their participation contributes to scientific progress. One approach, as highlighted before, involves providing summaries and thank you notes throughout and after the trial [40, 41]. By implementing such strategies and ensuring clear communication about, for example, the secondary use of genetic data and data sharing practices, we may help adolescents to become more aware about how their participation contributes to science and possible personal relevance of sharing and reanalysis of genetic data. The latter being of particular importance as adolescents might be more focused on the short‐rather than long‐term consequences of their decisions, especially regarding data security or insurrance [10, 42].

Offspring and cancer recurrence emerged as important topics for adolescents in our study. Consistent with previous findings, adolescents expressed both worries and hopes, for example that genetic testing would provide information about risks for themselves and their offspring, regarding these topics [42]. Studies among childhood cancer survivors indicate that these worries are common, regardless of whether survivors underwent genetic testing, suggesting that the worries reported by our participants are only partly attributable to genetic testing [43]. Previous research has emphasized the importance of discussing these worries to ensure survivors and their families feel adequately informed and supported [44].

Overall, the findings of this study, when interpreted in light of existing literature, suggest that adolescents with cancer undergoing genetic testing may have concerns typically associated with adulthood, such as worries about familial cancer or the possibility of cancer in their future children. This aligns with previous research showing that adolescents confronted with cancer report to have grown up faster than their healthy peers [45]. This signals a need to address these more mature aspects of genetic testing with adolescents, while carefully tailoring genetic counseling to each adolescent's cognitive and emotional development.

### 4.1 Implications

The results of this study suggest several areas for improving genetic counseling of adolescents with cancer. Specifically, the findings highlight the need for tailored educational strategies to help adolescents better understand relevant genetic concepts, as many of them could not correctly rate over half of the true/false statements presented to them. Particularly the limited understanding of adolescents on the scope and limitations of the genetic test suggests that this could receive more attention during informed consent. Recent studies suggest that visual ways of presenting information on genetic testing might be of value for adolescents [21, 33, 46]. Furthermore, we need to be critical on which knowledge adolescents truly need to make an informed decision on germline sequencing. The study also underscores the importance of discussing adolescents' hopes and worries regarding future cancer risks of family members and future offspring, as these topics seem most important to them. Since there may be overlap between the worries of adolescents related to genetics and worries related to fertility issues due to cancer treatment, addressing these topics together could be a possible approach [44, 48]. Finally, as some adolescents reported ongoing concerns after receiving negative results, support could be extended through routine follow‐up by healthcare professionals such as pediatric oncologists or case managers. Genetic information and related concerns may remain an ongoing topic of discussion, for example during transition to late effects clinics or when adolescents start thinking about having children, as these new phases may give rise to new questions [44].

### 4.2 Study Limitations

Several of the questionnaires used in this study were not originally developed or validated specifically for a pediatric oncology germline sequencing context. Although we made substantial efforts to adapt the items for this setting and to ensure they were comprehensible to adolescents, it's possible that some perspectives of the adolescents have not been fully captured. For example, dichotomized PAHC scores were used for descriptive and exploratory analyses; these cutoffs were originally developed for adults and have not been formally validated for adolescents. Although the PAHC was designed as a dichotomous screening instrument, dichotomization is known to reduce variance and may have limited statistical power to detect associations. The rarity of pediatric cancer limited the number of participants, potentially reducing the statistical power of our analyses and our ability to identify significant factors influencing knowledge or worries. Only a small number (*n* = 2) of participants received a positive test result, that is, learned that they had a genetic cancer predisposition syndrome. This is consistent with the literature, which shows that cancer predisposition syndromes explain only a minority of pediatric cancer cases [20, 47]. Due to the small number of cases, no meaningful conclusions can be drawn. Finally, participation in the REFLECT questionnaire study was limited, which may affect the generalizability of our findings. Adolescents who participated may differ from non‐participants in unmeasured factors, such as interest in genetic testing or willingness to engage in psychosocial research.

## 5 Conclusion

In the context of increasing use of germline genetic sequencing in pediatric oncology, our study underscores the importance of providing comprehensive genetic counseling to adolescents with cancer. Discussing their hopes and worries, not only regarding their own health, but also about future offspring, family members, and potential future patients, is crucial, as these topics are highly important to them. In addition to clear communication about the scope and limitations of genetic testing, healthcare professionals can play a role in supporting adolescents by addressing these concerns during regular follow‐up. Notably, we found no significant differences in perspectives based on gender.

## Author Contributions

conceptualization: SB, RW, MvdHE, MJ, MG, data curation: SB, HMS, formal analysis, SB, HMS, funding acquisition: SB, RW, MvdHE, MJ, MG, methodology: SB, HMS, RW, MvdHE, MJ, MG Project administration: SB, writing ‐ original draft: SB, writing ‐ review and editing: RW, MvdHE, MJ, MG, HMS, JB.

## Funding

KiKa, the Dutch Children Cancer Free Foundation, funded this study under Grant No. 403. PrediCT, the sequencing study that we collaborated with, also received funding from KiKa (Grant No. 355).

## Ethics Statement

Both the PrediCT and the REFLECT study were approved by the Medical Research Ethics Committee of the University Medical Center Utrecht (NL70480.041.20). Informed consent was obtained from all participating adolescents and, where applicable, from their legal guardian(s).

## Conflicts of Interest

All authors declare to have no competing interests.

## Supporting information


Supporting Information S1

## Data Availability

The data that support the findings of this study are available from the corresponding author upon reasonable request.

## References

[1] J. J. Bakhuizen , S. M. J. Hopman , M. I. Bosscha , et al. “Assessment of Cancer Predisposition Syndromes in a National Cohort of Children With a Neoplasm,” JAMA Network Open 6, no. 2 (February 2023): E2254157: [Internet], 10.1001/jamanetworkopen.2022.54157. 36735256 PMC9898819

[2] G. M. Brodeur , K. E. Nichols , S. E. Plon , J. D. Schiffman , and D. Malkin , “Pediatric Cancer Predisposition and Surveillance: An Overview, and a Tribute to Alfred G. Knudson Jr,” Clinical Cancer Research 23, no. 11 (2017): e1–e5, 10.1158/1078-0432.ccr-17-0702. 28572261 PMC5553563

[3] F. A. M. Postema , S. M. J. Hopman , R. C. Hennekam , and J. H. M. Merks , “Consequences of Diagnosing a Tumor Predisposition Syndrome in Children With Cancer: A Literature Review,” Pediatric Blood and Cancer 65, no. 1 (January 2018): e26718: [Internet], http://doi.wiley.com/10.1002/pbc.26718. 10.1002/pbc.26718 28834056

[4] J. Wise , “Genome Sequencing of Children Promises a New Era in Oncology,” BMJ (January 2019): l105: [Internet], https://www.bmj.com/lookup/doi/10.1136/bmj.l105. 30622102 10.1136/bmj.l105

[5] Netherlands Cancer Registry (NCR) . Netherlands Comprehensive Cancer Organisation (IKNL) [Internet]. 2022 [cited 2024 Aug 20], www.cijfersoverkanker.nl.

[6] Children, Teenagers and Young Adults UK Cancer Statistics Report 2021 . 2021, [Internet], https://digital.nhs.uk/binaries/content/assets/website‐assets/national‐disease‐registration‐service/ctya/uk_ctya_cancer_statistics_report_2021_040321.pdf.

[7] America’s Children and the Environment: Health – Childhood cancer . 2022, [Internet], https://www.epa.gov/americaschildrenenvironment/health‐childhood‐cancer.

[8] J. Pervola , M. F. Myers , M. L. McGowan , and C. A. Prows , “Giving Adolescents a Voice: The Types of Genetic Information Adolescents Choose to Learn and Why,” Genetics in Medicine 21, no. 4 (2019): 965–971, 10.1038/s41436-018-0320-1. 30369597 PMC10445294

[9] A. Pichini , C. Shuman , K. Sappleton , M. Kaufman , D. Chitayat , and R. Babul‐Hirji , “Experience With Genetic Counseling: The Adolescent Perspective,” Journal of Genetic Counseling 25, no. 3 (2016): 583–595, 10.1007/s10897-015-9912-y. 26573304

[10] P. Grootens‐Wiegers , I. M. Hein , J. M. van den Broek , and M. C. de Vries , “Medical Decision‐Making in Children and Adolescents: Developmental and Neuroscientific Aspects,” BMC Pediatrics 17, no. 1 (December 2017): 120: [Internet], 10.1186/s12887-017-0869-x. 28482854 PMC5422908

[11] C. Lewis , B. S. Loe , C. Sidey‐Gibbons , C. Patch , L. S. Chitty , and S. C. Sanderson , “Development of a Measure of Genome Sequencing Knowledge for Young People: The Kids‐KOGS,” Clinical Genetics 96, no. 5 (November 2019): 411–417: [Internet], 10.1111/cge.13607. 31323115 PMC6851564

[12] M. Machová and E. Ehler , “Secondary School Students’ Misconceptions in Genetics: Origins and Solutions,” Journal of Biological Education 57, no. 3 (May 2023): 633–646: [Internet], 10.1080/00219266.2021.1933136.

[13] B. C. McGill , C. E. Wakefield , J. Vetsch , et al. “Children and Young People’s Understanding of Inherited Conditions and Their Attitudes Towards Genetic Testing: A Systematic Review,” Clinical Genetics 95, no. 1 (January 2019): 10–22: [Internet], 10.1111/cge.13253. 29574695

[14] J. Vetsch , C. E. Wakefield , M. Warby , et al. “Cancer‐Related Genetic Testing and Personalized Medicine for Adolescents: A Narrative Review of Impact and Understanding,” Journal of Adolescent and Young Adult Oncology 7, no. 3 (June 2018): 259–262: [Internet], 10.1089/jayao.2017.0102. 29336661

[15] R. Forbes Shepherd , A. Werner‐Lin , L. A. Keogh , M. B. Delatycki , and L. E. Forrest , “‘I Need to Know if I’M Going to Die Young’: Adolescent and Young Adult Experiences of Genetic Testing for Li–Fraumeni Syndrome,” Journal of Psychosocial Oncology 39, no. 1 (2021): 54–73: [Internet], 10.1080/07347332.2020.1768199. 32449501

[16] C. E. Wakefield , K. Hetherington , E. G. Robertson , et al. “Hopes, Concerns, Satisfaction and Regret in a Precision Medicine Trial for Childhood Cancer: A Mixed‐Methods Study of Parent and Patient Perspectives,” British Journal of Cancer 129, no. 10 (November 2023): 1634–1644: [Internet], 10.1038/s41416-023-02429-1. 37726477 PMC10645918

[17] J. M. Marron , S. G. DuBois , J. G. Bender , et al. “Patient/Parent Perspectives on Genomic Tumor Profiling of Pediatric Solid Tumors: The Individualized Cancer Therapy (Icat) Experience,” Pediatric Blood and Cancer 63, no. 11 (2016): 1974–1982, 10.1002/pbc.26137. 27429135 PMC5611837

[18] J. D. Hunter , K. Hetherington , E. Courtney , et al. “Parents’ and Patients’ Perspectives, Experiences, and Preferences for Germline Genetic or Genomic Testing of Children With Cancer: A Systematic Review,” Genetics in Medicine 26, no. 9 (June 2024): 101197: [Internet], 10.1016/j.gim.2024.101197. 38943478

[19] C. E. Wakefield , L. V. Hanlon , K. M. Tucker , et al. “The Psychological Impact of Genetic Information on Children: A Systematic Review,” Genetics in Medicine 18, no. 8 (August 2016): 755–762: [Internet], 10.1038/gim.2015.181. 26741411

[20] J. J. Bakhuizen , F. van Dijk , M. J. Koudijs , et al. “Comparison of Clinical Selection‐Based Genetic Testing With Phenotype‐Agnostic Extensive Germline Sequencing to Diagnose Genetic Predisposition in Children With Cancer: A Prospective Diagnostic Study,” Lancet Child and Adolescent Health 8, no. 10 (October 2024): 751–761: [Internet], 10.1016/s2352-4642(24)00144-5. 39159644

[21] S. B. B. Bon , R. H. P. Wouters , J. J. Bakhuizen , M. C. J. Jongmans , M. M. van den Heuvel‐Eibrink , and M. A. Grootenhuis , “Experiences of Pediatric Cancer Patients (Age 12–18 Years) With Extensive Germline Sequencing for Cancer Predisposition: A Qualitative Study,” European Journal of Human Genetics 32, no. 5 (February 2024): 567–575: [Internet], 10.1038/s41431-024-01565-3. 38409533 PMC11061193

[22] S. B. B. Bon , R. H. P. Wouters , J. J. Bakhuizen , et al. “Parents’ Experiences With Sequencing of all Known Pediatric Cancer Predisposition Genes in Children With Cancer,” Genetics in Medicine 27, no. 1 (September 2024): 101250: [Internet], 10.1016/j.gim.2024.101250. 39244644

[23] W. Eijzenga , E. M. A. Bleiker , D. E. E. Hahn , et al. “Psychosocial Aspects of Hereditary Cancer (PAHC) Questionnaire: Development and Testing of a Screening Questionnaire for Use in Clinical Cancer Genetics,” Psycho‐Oncology 23, no. 8 (August 2014): 862–869: [Internet], 10.1002/pon.3485. 24443031

[24] C. V. Fernandez , E. Bouffet , D. Malkin , et al. “Attitudes of Parents Toward the Return of Targeted and Incidental Genomic Research Findings in Children,” Genetics in Medicine 16, no. 8 (August 2014): 633–640: [Internet], 10.1038/gim.2013.201. 24434691

[25] J. A. Oberg , J. Ruiz , T. Ali‐Shaw , et al. “Whole‐Genome and Whole‐Exome Sequencing in Pediatric Oncology: An Assessment of Parent and Young Adult Patient Knowledge, Attitudes, and Expectations,” JCO Precision Oncology 2 (December 2018): 1–11: [Internet], https://ascopubs.org/doi/10.1200/PO.17.00104. 10.1200/PO.17.00104 PMC7446494 32913997

[26] J. C. Brehaut , A. M. O’Connor , T. J. Wood , et al. “Validation of a Decision Regret Scale,” Medical Decision Making 23, no. 4 (July 2003): 281–292: [Internet], 10.1177/0272989x03256005. 12926578

[27] A. O’Connor , “User Manual ‐ Decision Regret Scale [Internet],” Ohri 1 (2003): 1–3: [cited 2024 Jun 26], http://decisionaid.ohri.ca/docs/develop/User_Manuals/UM_Regret_Scale.pdf.

[28] Stiggelbout A. M. , Pieterse A. H. Decision Regret Scale Dutch Translation [Internet]. (2010) [cited 2019 Apr 1], https://decisionaid.ohri.ca/docs/develop/Tools/Regret_Scale_Dutch.pdf.

[29] M. M. Becerra‐Perez , M. Menear , S. Turcotte , M. Labrecque , and F. Légaré , “More Primary Care Patients Regret Health Decisions if They Experienced Decisional Conflict in the Consultation: A Secondary Analysis of a Multicenter Descriptive Study,” BMC Family Practice 17, no. 1 (December 2016): 1–11: [Internet], 10.1186/s12875-016-0558-0. 27832752 PMC5103443

[30] M. F. Myers , L. J. Martin , and C. A. Prows , “Adolescents’ and Parents’ Genomic Testing Decisions: Associations With Age, Race, and Sex,” Journal of Adolescent Health 66, no. 3 (March 2020): 288–295: [Internet], 10.1016/j.jadohealth.2019.08.028. PMC7007858 31685375

[31] T. L. Beauchamp and J. F. Childress , “Respect for Autonomy,” in Principles of Biomedical Ethics, 8th ed. (Oxford University Press, 2019).

[32] I. A. Holm , T. W. Yu , and S. Joffe , “From Sequence Data to Returnable Results: Ethical Issues in Variant Calling and Interpretation,” Genetic Testing and Molecular Biomarkers 21, no. 3 (March 2017): 178–183: [Internet], 10.1089/gtmb.2016.0413. 28306396 PMC5367907

[33] J. M. Gereis , K. Hetherington , E. G. Robertson , et al. “Parents’ and Adolescents’ Perspectives and Understanding of Information About Childhood Cancer Precision Medicine,” Cancer 129, no. 22 (2023): 3645–3655, 10.1002/cncr.34914. 37376781

[34] L. K. Tolusso , K. Collins , X. Zhang , J. R. Holle , C. A. Valencia , and M. F. Myers , “Pediatric Whole Exome Sequencing: An Assessment of Parents’ Perceived and Actual Understanding,” Journal of Genetic Counseling 26, no. 4 (August 2017): 792–805: [Internet], 10.1007/s10897-016-0052-9. 27987066

[35] J. M. Marron , E. Quach , Y. Pikman , K. A. Janeway , and J. W. Mack , “Participant Hopes and Expectations Regarding Outcomes of Genomic Sequencing Research in Pediatric Oncology,” Journal of Clinical Oncology 37, no. 15 (May 2019): 10020: [Internet], http://ascopubs.org/doi/10.1200/JCO.2019.37.15_suppl.10020.

[36] N. S. Y. Liang , S. Adam , A. M. Elliott , et al. “After Genomic Testing Results: Parents’ Long‐Term Views,” Journal of Genetic Counseling 31, no. 1 (2022): 82–95, 10.1002/jgc4.1454. 34165210

[37] S. Van Hoyweghen , K. Bm Claes , R. de Putter , et al. “The Psychological Impact of Genetic Testing in Childhood Cancer: A Systematic Review,” Psycho‐Oncology 33, no. 1 (January 2024): e6279: [Internet], https://onlinelibrary.wiley.com/doi/10.1002/pon.6279. 38282231 10.1002/pon.6279

[38] D. Skinner , K. A. Raspberry , and M. King , “The Nuanced Negative: Meanings of a Negative Diagnostic Result in Clinical Exome Sequencing,” Sociology of Health and Illness 38, no. 8 (November 2016): 1303–1317: [Internet], 10.1111/1467-9566.12460. 27538589 PMC5089912

[39] S. Van Hoyweghen , K. B. M. Claes , R. de Putter , et al. “Family‐Level Impact of Germline Genetic Testing in Childhood Cancer: A Multi Family Member Interview Analysis,” Cancers (Basel) 17, no. 3 (February 2025): 517: [Internet], 10.3390/cancers17030517. 39941887 PMC11816119

[40] K. O. Zimmerman , B. Perry , E. Hanlen‐Rosado , et al. “Developing Lay Summaries and Thank You Notes in Paediatric Pragmatic Clinical Trials,” Health Expectations 25, no. 3 (June 2022): 1029–1037: [Internet], 10.1111/hex.13448. 35246906 PMC9122399

[41] C. V. Fernandez , J. Gao , C. Strahlendorf , et al. “Providing Research Results to Participants: Attitudes and Needs of Adolescents and Parents of Children With Cancer,” Journal of Clinical Oncology 27, no. 6 (February 2009): 878–883: [Internet], 10.1200/jco.2008.18.5223. 19164211 PMC2668636

[42] C. Lewis , J. Hammond , M. Hill , et al. “Young People’s Understanding, Attitudes and Involvement in Decision‐Making About Genome Sequencing for Rare Diseases: A Qualitative Study With Participants in the UK 100, 000 Genomes Project,” European Journal of Medical Genetics 63, no. 11 (November 2020): 104043: [Internet], 10.1016/j.ejmg.2020.104043. 32835846

[43] A. Maas , A. Westerweel , H. Maurice‐Stam , et al. “The Prevalence and Associated Factors of Cancer‐Related Worries in Adult Survivors of Childhood Cancer: A Systematic Review,” Psycho‐Oncology 34, no. 2 (2025): e70101, 10.1002/pon.70101. 39947662 PMC11825232

[44] J. Vetsch , C. E. Wakefield , K. M. Tucker , et al. “Genetics‐Related Service and Information Needs of Childhood Cancer Survivors and Parents: A mixed‐methods Study,” European Journal of Human Genetics 28, no. 1 (2020): 6–16, 10.1038/s41431-019-0481-7. 31363185 PMC6906423

[45] Dattilo T. M. , Olshefski R. S. , Nahata L. , Hansen‐Moore J. A. , Gerhardt C. A. , Lehmann V. “Growing Up After Childhood Cancer: Maturity and Life Satisfaction in Young Adulthood.” Supportive Care in Cancer [Internet]. (November 2021) [cited 2025 Jun 29];29(11):6661–6668, 10.1007/s00520-021-06260-3. 33961121 PMC8464568

[46] M. Sabatello , Y. Chen , S. C. Sanderson , W. K. Chung , and P. S. Appelbaum , “Increasing Genomic Literacy Among Adolescents,” Genetics in Medicine 21, no. 4 (2019): 994–1000: [Internet], 10.1038/s41436-018-0275-2. 30214064 PMC6417977

[47] J. J. Bakhuizen , F. Bourdeaut , K. A. W. Wadt , C. P. Kratz , M. C. J. Jongmans , and N. Waespe , Genetic Testing for Childhood Cancer Predisposition Syndromes: Controversies and Recommendations from the SIOPE Host Genome Working Group Meeting 2022 Vol. 4 EJC Paediatric Oncology, (December 2024): [Internet], 10.1016/j.ejcped.2024.100176.

[48] F. Filippi , F. Peccatori , S. Manoukian , et al. “Fertility Counseling in Survivors of Cancer in Childhood and Adolescence: Time for a Reappraisal? Cancers (Basel) [Internet],” Cancers 13, no. 22 (November 2021): 5626, 10.3390/cancers13225626. 34830781 PMC8615855

