# Supplementary material for: Adolescents' Experiences With Sequencing for Genetic Predisposition in Pediatric Cancer: A Quantitative Study
Source: Psychooncology. 2026 Apr 7;35(4):e70458. doi: 10.1002/pon.70458 (PMC13057058; doi:10.1002/pon.70458)
Supplement: Supplementary file 1 — Supporting Information S1 [file PON-35-e70458-s001.docx]

Supplementary tables

Supplementary table 1. Linear regression model assessing the association between adolescent’ age and knowledge score

| **Variable** | **B** | **Std. Error** | **Beta** | **t** | **Sig.** | **95% CI for B** |
| --- | --- | --- | --- | --- | --- | --- |
| Age of Child | 1.101 | 0.356 | 0.419 | 3.093 | 0.003 | [0.373, 1.829] |

Supplementary table 2. The association of demographical and medical characteristics with the knowledge score

|  | N | Mean | SD | p-value |
| --- | --- | --- | --- | --- |
| *Gender* |  |  |  | 0.77^a^ |
| Male | 25 | 11.24 | 5.13 |  |
| Female | 22 | 10.82 | 4.74 |  |
| *Referred for genetic consultation* |  |  |  | 0.55^a^ |
| No | 32 | 11.38 | 4.90 |  |
| Yes | 15 | 10.33 | 4.98 |  |
| *Primary diagnosis* |  |  |  | 0.95^b^ |
| Hematologic | 17 | 11.60 | 5.83 |  |
| CNS | 17 | 11.00 | 4.50 |  |
| Solid non-CNS | 13 | 11.64 | 5.35 |  |
| *Highest educational level parents* |  |  |  | 0.66^b^ |
| Low | 3 | 8.00 | 0.00 |  |
| Medium | 13 | 10.75 | 3.90 |  |
| High | 31 | 11.47 | 5.88 |  |

^a^ according to independent-sample T-test

^b^ according to one-way ANOVA

Supplementary table 3. The association of demographical and medical characteristics with with being quite a bit/very much worried on one or more PAHC questions at the first measurement (N=47)

| **Variable** | Not at all/ a little worried  % (N) | Quite a bit/very much worried  % (N) | p-value ^a^ |
| --- | --- | --- | --- |
| *Gender* |  |  | 0.49 |
| Male | 50.0 (11) | 50.0 (11) |  |
| Female | 60.0 (15) | 40.0 (10) |  |
| *Referred for genetic consultation* |  |  | 0.28 |
| No | 50.0 (16) | 50.0 (16) |  |
| Yes | 66.7 (10) | 33.3 (5) |  |
| *Primary diagnosis* |  |  | 0.47 |
| Hematologic | 47.1 (8) | 52.9 (9) |  |
| CNS | 52.9 (9) | 47.1 (8) |  |
| Solid non-CNS | 69.2 (9) | 30.8 (4) |  |
| *Highest educational level parents* |  |  | 0.71 |
| Low | 33.3 (1) | 66.7 (2) |  |
| Medium | 53.8 (7) | 46.2 (6) |  |
| High | 58.1 (18) | 41.9 (13) |  |

^a^ according to Chi-square test

Supplementary table 4. Binary logistic model assessing the association between age and being quite a bit/very much worried on one or more PAHC questions at the first measurement.

| **Variable** | **B** | **S.E.** | **Wald** | **df** | **Sig.** | **Exp(B)** | **95% CI for Exp(B)** |
| --- | --- | --- | --- | --- | --- | --- | --- |
| Age in years | 0.010 | 0.159 | 0.004 | 1 | 0.947 | 1.011 | 0.740 - 1.379 |

Supplementary table 5. Crosstabulation and Chi-Square tests of various variables and quite a bit or very much worries on one or more PAHC questions at the second measurement.

| **Variable** | Not at all/ a little worried  % (N) | Quite a bit/very much worried  % (N) | p-value ^a^ |
| --- | --- | --- | --- |
| *Gender* |  |  | 0. |
| Male | . () | . () |  |
| Female | . () | . () |  |
| *Referred for genetic consultation* |  |  | 0. |
| No | . () | . () |  |
| Yes | . () | . () |  |
| *Primary diagnosis* |  |  | 0. |
| Hematologic | . () | . () |  |
| CNS | . () | . () |  |
| Solid non-CNS | . () | . () |  |
| *Highest educational level parents* |  |  | 0. |
| Low | . () | . () |  |
| Medium | . () | . () |  |
| High | . () | . () |  |

^a^ according to Chi-square test

Supplementary table 6. Binary logistic model assessing the association between age and being quite a bit/very much worried on one or more PAHC questions at the second measurement.

| **Variable** | **B** | **S.E.** | **Wald** | **df** | **Sig.** | **Exp(B)** | **95% CI for Exp(B)** |
| --- | --- | --- | --- | --- | --- | --- | --- |
| Age of Child | 0.236 | 0.200 | 1.391 | 1 | 0.238 | 1.266 | 0.850 - 1.884 |
